# Leonurine Alleviates DSS‐Induced Colitis in Mice by Regulating Pancreatic Secretion Pathway and Gut Microbiota

**DOI:** 10.1155/jimr/6626309

**Published:** 2025-10-10

**Authors:** TingTing Cao, Ying Wang, Juan Zhang, Wei Song, WeiJie Dai, GuoZhong Ji, Poorani Gurumallesh

**Affiliations:** ^1^ Department of Gastroenterology, The Affiliated Huaian No. 1 People’s Hospital of Nanjing Medical University, Huai’an, 223300, China, njmu.edu.cn; ^2^ Department of Pharmacy, The Affiliated Huai’an Hospital of Xuzhou Medical University and the Second People’s Hospital of Huai’an, Huai’an, 223300, China, xzmc.edu.cn; ^3^ Medical Centre for Digestive Diseases, The Second Affiliated Hospital of Nanjing Medical University, Nanjing, 210011, China, njmu.edu.cn

**Keywords:** colitis, gut microbiota, leonurine, pancreatic secretion pathway

## Abstract

Ulcerative colitis (UC) is a chronic, recurrent inflammatory bowel disease (IBD). Leonurine is an active component in *Leonurus japonicus*, involved in several processes such as inflammation, oxidation, and other processes. This study found that symptoms of colitis induced by 3% dextran sulfate sodium (DSS) solution in C57BL/6 mice were significantly alleviated after administration of leonurine, in terms of reduced body weight, shortened colon length, disease activity index (DAI), and colonic pathological damage. The expression of the tight junction (TJ) protein claudin‐1 and occludin markedly increased, the levels of tumor necrosis factor‐alpha (TNF‐α) and interleukin‐1β (IL‐1β) significantly decreased. Findings from transmission electron microscopy (TEM) and intestinal permeability assessment experiments indicated that leonurine ameliorates the intestinal barrier. Leonurine regulated the pancreatic secretion pathway, significantly reduced the expression levels of *Cela2a* and *Cela3b*, and clearly decreased the abundance of Rikenellaceae_RC9_gut_group, UBA1819, *Enterococcus*, and *Oscillibacter*. We proposed that leonurine may improve DSS‐induced colitis by regulating the pancreatic secretion pathway, modulating the gut microbiota, and improving intestinal barrier, potentially becoming a candidate for the treatment of UC.

## 1. Introduction

Ulcerative colitis (UC) is a major form of inflammatory bowel disease (IBD), characterized by inflammation that is diffuse, superficial, and limited to the mucosa [1]. The worldwide incidence of UC is rising, but the pathogenesis is complex and incompletely understood. Despite advances in treatment, only approximately 40% of patients achieve clinical remission at the end of 1 year, prompting the exploration of new treatment modalities [2].

*Leonurus japonicus*, a plant of the Lamiaceae family, contains a variety of active components, among which leonurine has received considerable attention. Leonurine was extracted from the fresh leaves of *Leonurus* [3]. Its structure is characterized by a guanidino, an n‐butyl, and a syringate, as shown in Figure 1A. Accumulating evidence has indicated that leonurine regulates many biological processes (BPs), such as oxidation processes, inflammatory responses, apoptosis, and lipid metabolism [4]. Leonurine can relieve acute kidney injury, myocarditis, pneumonia, and rheumatoid arthritis [5–8]. Currently, there is limited research on the use of leonurine in the treatment of colitis. Dextran sulfate sodium (DSS)‐induced colitis is a well‐characterized and widely used model of acute colitis that is similar to human UC [9]. Therefore, in this study, we aimed to evaluate the effects of leonurine on DSS‐induced colitis in mice and clarify the underlying mechanisms.

Figure 1Leonurine attenuates the symptoms of DSS‐induced colitis. (A) The molecular structure of leonurine. (B) Body weight changes of each group. (C) DAI scores of each group. (D) The macroscopic pictures of colons. (E) Colon lengths of each group at 8d. (F) The histologic score. (G) HE‐stained pictures of colon tissues in three groups (scale bars: 100 μm), the widespread epithelial loss (black arrow), crypt damage (red arrow), and massive inflammatory cellular infiltration (blue arrow). Values were plotted as mean ± SEM, *n* = 6.  ^∗^ *p* < 0.05,  ^∗∗^ *p* < 0.01,  ^∗∗∗^ *p* < 0.001.

**Figure:**
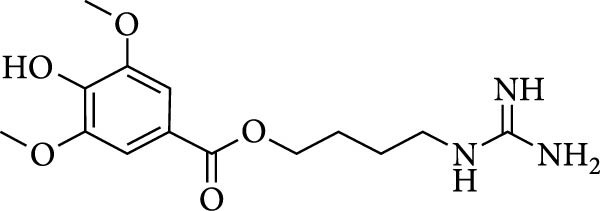
(A)

**Figure:**
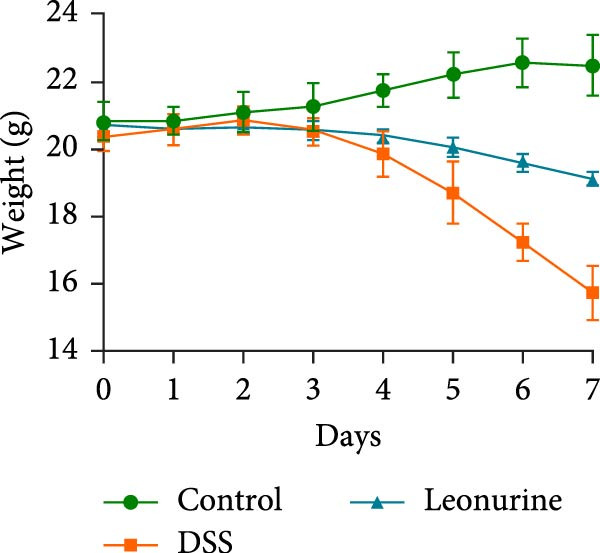
(B)

**Figure:**
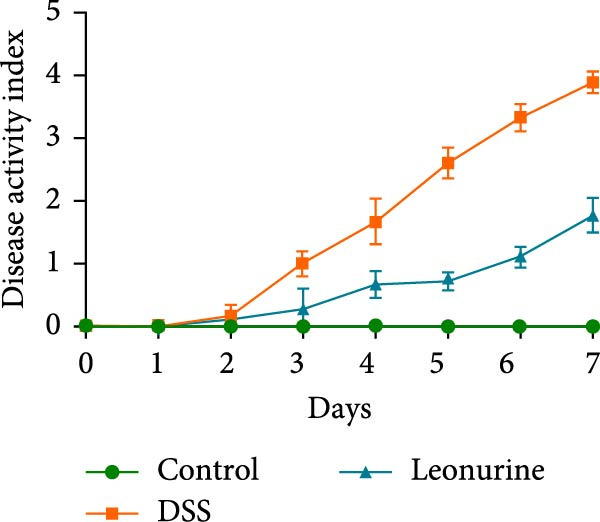
(C)

**Figure:**
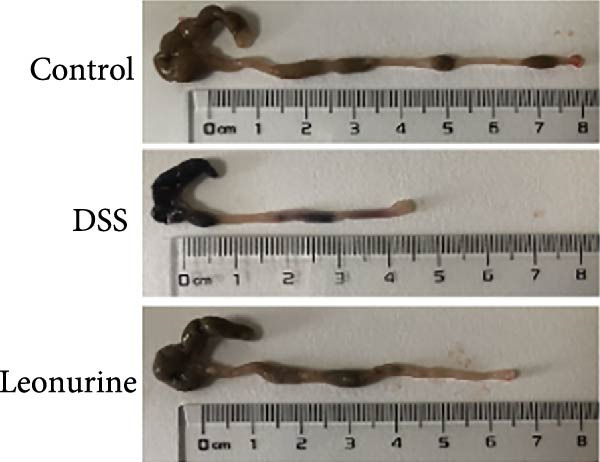
(D)

**Figure:**
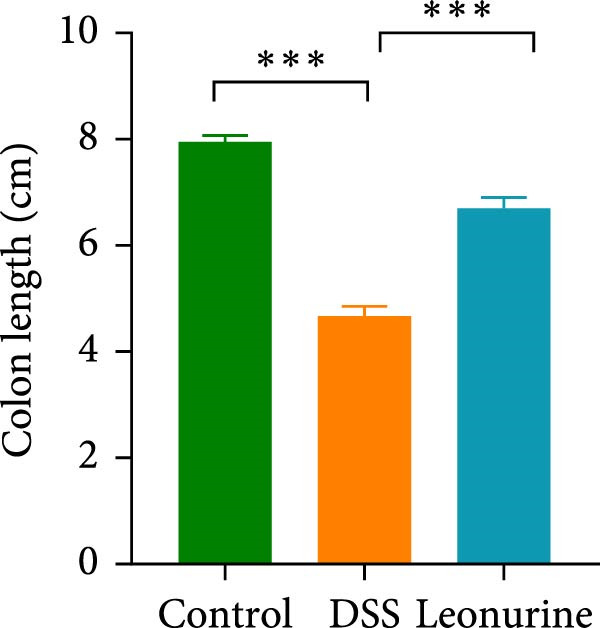
(E)

**Figure:**
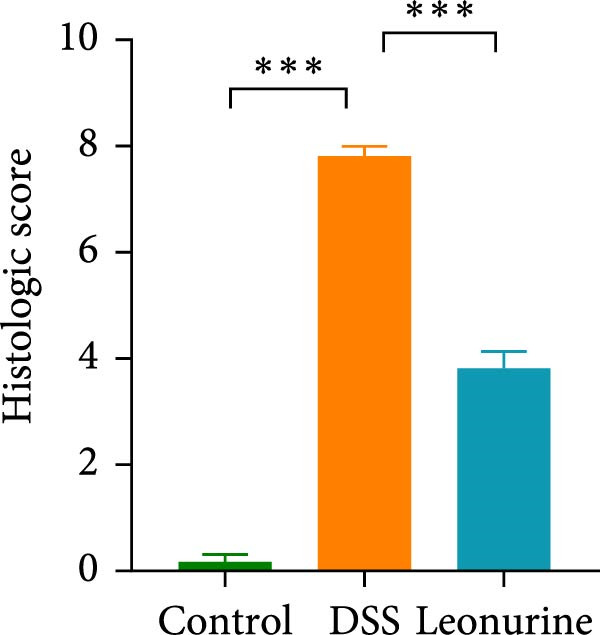
(F)

**Figure:**
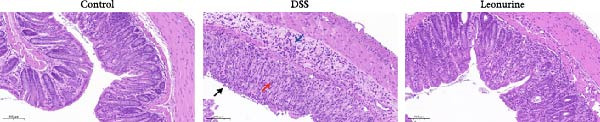
(G)

## 2. Materials and Methods

### 2.1. Animal Experiments

Male C57BL/6 mice (weight: 19–21 g and age: 6–8 weeks) were purchased from Hangzhou Ziyuan Laboratory Animal Technology Co., Ltd. (China). All mice were maintained at the Center for Experimental Animals of the Affiliated Huai’an No. 1 People’s Hospital of Nanjing Medical University, and housed at a temperature of 21 ± 2°C and humidity of 45 ± 10% in a specific pathogen‐free environment with a 12 h light/dark cycle and given free access to a standard laboratory diet and water. All procedures involving animals were approved by the Animal Ethics Committee of Nanjing Medical University.

After acclimatization for at least 1 week, the mice were randomly divided into three groups (six mice per group) as follows: control group: mice were fed normal drinking water for 7 days; DSS group: mice were fed 3% DSS in drinking water for 7 days to induce acute colitis; leonurine group: mice were fed 3% DSS in drinking water and orally gavaged with leonurine at 30 mg/kg/d for 7 days, a dose selected based on previous studies demonstrating its efficacy in ameliorating mice colitis [10]. On day 8, all mice in this experiment were euthanized, blood samples were obtained from the heart, and fresh feces and colon tissues were collected for further analysis after measurement of colon length. DSS (molecular weight: 36–50 kDa) was purchased from MP Biomedicals, USA. Leonurine was purchased from Chengdu Herbpurify Co., Ltd (Sichuan, China).

### 2.2. Disease Activity Index (DAI)

The DAI scores were calculated and expressed as the average of the following three parameters: weight decrease (1 : 1%–5%, 2 : 5%–10%, 3 : 10%–15%, and 4: ≥15%), stool consistency (0: normal, 2: loose stools, and 4: watery diarrhea), and hematochezia (0: no bleeding, 2: slight bleeding, and 4: gross bleeding).

### 2.3. Histopathological Analysis

The distal 1.5 cm of the colon was obtained and fixed in 4% paraformaldehyde, dehydrated with gradient alcohol, and embedded in paraffin. Five‐μm‐thick sections were stained. The histological score was calculated according to inflammatory cell infiltration and the degree of epithelial loss.

### 2.4. Immunohistochemical Staining

The colon tissue sections were deparaffinized and hydrated, placed in an antigen repair solution, and boiled for antigen repairing. Claudin‐1 and occludin primary antibodies were incubated overnight at 4°C. Then, the sections were rinsed three times with PBS and incubated with secondary antibodies labeled with horseradish peroxidase at room temperature for 1 h. DAB was used for developing the color, and the slides were mounted after hematoxylin counterstaining for microscopic examination. The final image results were obtained through panoramic scanning, and the scanning results were quantitatively analyzed using the pathological image analysis software Halo. The different staining signal conditions of each cell within the field of view were identified and analyzed, and the percentage of claudin‐1 and occludin positive cells in the total cell count of the sample was statistically determined.

### 2.5. Transmission Electron Microscopy (TEM) Analysis

The 5 mm fresh intestinal tissue fragments were rinsed with PBS, then fixed in 2.5% glutaraldehyde at 4°C for 4 h. After rinsing with PBS, the fragments were fixed in 1% osmium tetroxide at room temperature for 2 h. The samples were then sectioned using an ultramicrotome, and images were obtained using a transmission electron microscope.

### 2.6. ELISA

Whole blood was allowed to coagulate at room temperature for 10–20 min, then centrifuged at 3000 rpm for 20 min to separate the serum. Inflammatory cytokines tumor necrosis factor‐alpha (TNF‐α) and interleukin‐1β (IL‐1β) levels were determined using ELISA kits (Enzyme‐linked Biotechnology Co., Ltd, Shanghai, China) according to the manufacturer’s instructions.

### 2.7. Intestinal Permeability Assessment

On day 8 of the experiment, the mice were fasted for 4 h. Subsequently, they were administered FITC‐dextran 4000 (Yeasen, Shanghai, China) via oral gavage at a dose of 500 mg/kg. Three hours after gavage, the mice were euthanized, and blood samples were collected. The blood samples were then centrifuged, and the plasma was harvested. A multimode microplate reader was used to analyze the fluorescence intensity of the samples (excitation wavelength: 490 nm and emission wavelength: 525 nm). The concentration of FITC‐dextran was determined using a standard curve.

### 2.8. Transcriptome Sequencing

Four mice from each group were randomly selected and 1 cm‐long inflammatory colon tissues were collected for sequencing. Total RNA was isolated from the colon tissues with TRIzol Reagent in accordance with the manufacturer’s instructions. Following the manufacturer’s instructions (Illumina, San Diego, CA, USA), RNA purification, reverse transcription, and library construction were performed. Paired‐end RNA‐seq sequencing library was generated on the Illumina NovaSeq 6000 sequencer (PE150). The raw reads were deposited into the NCBI Sequence Read Archive (SRA) database (Accession Number: PRJNA1124231).

Quality control was performed on the raw paired‐end reads using fastp [11] with default parameters. HISAT2 [12] software was used to separately align the clean reads to the reference genome. The mapped reads of each sample were assembled by StringTie via a reference‐based approach [13]. Differentially expressed genes (DEGs) were analyzed by DESeq2 [14], and those with *p*‐adjusted‐values less than 0.05 were considered as significant DEGs. In addition, Gene Ontology (GO) functional enrichment and Kyoto Encyclopedia of Genes and Genomes (KEGG) enrichment analysis were performed using the R package ClusterProfiler.

### 2.9. qRT‐PCR

cDNA was synthesized with 2 μg RNA by the PrimeScript RT Reagent Kit with gDNA Eraser (TaKaRa). Based on the target cDNA sequences, gene‐specific primers for (qRT‐PCR) analysis were designed using Primer 5.0 (the primer sequences are provided in Table 1). The *ACTIN* was performed as the internal reference gene. The qRT‐PCR reaction was performed using SYBR Premix Ex TaqTM II (Tli RNaseH Plus) and an ABI 7500 Real‐time Detection System (Thermo Fisher Scientific, USA). The PCR reaction conditions were 95°C for 10 min, 40 cycles of 95°C for 15 s, and 60°C for 60 s. The qRT‐PCRs were run in four biological replicates with three technical replicates and the data were represented as the mean ± SD (*n* = 4) and assessed with Student’s *t*‐test. The relative gene expression was calculated using the 2^−*△△*CT^ algorithm (Pfaffl, 2001).

**Table 1:** The primers information of genes in this study.

Gene name	Primer sequences	Product length (bp)
*Cela2a*	F: TGAGCAAGAACATCCAGACAG	167
	R: GCTGGAGCAGGTGGCATA	—

*Cela3b*	F: GGACAGCCTTCCCACAAC	160
	R: CCGCAGTCAGAACCCAGT	—

*ACTIN*	F: CCATCTACGAGGGCTATGCT	150
	R: CTTTGATGTCACGCACGATT	—

### 2.10. Gut Microbiota Analysis

The CTAB method was used to extract the total genomic DNA from the fecal samples. The V3‐V4 hypervariable region of the bacterial 16S rRNA gene was amplified with primer pairs 338F (5’‐ACTCCTACGGGAGGCAGCAG‐3’) and 806R(5’‐GGACTACHVGGGTWTCTAAT‐3’) by an ABI GeneAmp 9700 PCR thermocycler (ABI, CA, USA). The PCR amplification of the 16S rRNA gene was run as follows: initial denaturation at 95°C for 3 min, followed by 29 cycles of denaturation at 95°C for 30 s, annealing at 53°C for 30 s and extension at 72°Cfor 45 s, with single extension at 72°C for 10 min, and ending at 4°C. The PCR reaction mixture contained 10 μL 2xPro taq, 0.8 μL forward primer (5 μM), 0.8 μL reverse primer (5 μM), 10 ng template DNA, and finally ddH_2_O up to 20 μL. PCR reactions were conducted in triplicate. The PCR product was purified using 2% agarose gel and the DNA was recovered using the AxyPrep DNA Gel Extraction Kit (Axygen Biosciences, Union City, CA, USA) according to the manufacturer’s instructions and quantified using a Quantus Fluorometer (Promega, USA). Purified amplicons were pooled in equimolar ratios and paired‐end sequenced on an Illumina MiSeq PE300 platform (Illumina, San Diego, USA).

The unoise3 algorithm was used to denoised the high‐quality sequences by usearch11. The denoised sequences are called amplicon sequence variants (ASVs). All sequences were classified into different taxonomic groups by running a BLAST search against the SILVA138 database. Rarefaction curves were made using QIIME (v1.8.0). The alpha index was calculated based on the ASV information. Based on the relative abundance and taxonomic annotation results, bar plot diagram analyses were performed using R (v3.6.0) software. To test the similarity between different samples, clustering analyses and principal component analyses were performed using R (v3.6.0) based on the ASV information from each sample [15].

### 2.11. Statistical Analysis

Statistical analysis was performed using GraphPad Prism 9.4.1. Data were presented as mean ± SEM. Differences among groups were assessed by one‐way ANOVA followed by Tukey test. A *p*‐value < 0.05 was considered statistically significant.

## 3. Results

### 3.1. Leonurine Attenuates the Symptoms of DSS‐Induced Colitis

To further validate the therapeutic effects of leonurine, C57BL/6 mice were fed 3% DSS solution for 7 consecutive days. From day 3 onward, the DSS group lost more body weight (Figure 1B), and had higher DAI scores than the control group (Figure 1C). Moreover, the length of the colon in DSS mice was significantly shorter than in control mice (Figure 1D). However, leonurine administration significantly improved body weight loss, reduced DAI scores, and recovered the shortened colon length in the DSS group. Hematoxylin and eosin (H&E) staining revealed the widespread epithelial loss, crypt damage, and massive inflammatory cellular infiltration in DSS group, that resulted in higher histological scores, whereas treatment with leonurine had beneficial effects, significantly attenuating colon injury and consequently decreasing the histological scores (Figure 1E,F).

### 3.2. Leonurine Protects the Intestinal Barrier and Decreases the Levels of Proinflammatory Cytokines in DSS‐Induced Colitis Models

Claudin‐1 and occludin were measured to analyze the effect of leonurine on the intestinal barrier. Immunohistochemistry showed that their expression was decreased in the DSS group, whereas leonurine administration significantly increased its expressions in the colon tissue (Figure 2A,C,D). As illustrated in the TEM image (Figure 2B), tight junctions (TJs) beneath the microvilli in the colon tissue of the control group were continuous, intact, and narrow. In contrast, TJ in the DSS group appeared blurred and exhibited widened gaps. Notably, the leonurine group displayed a more intact and clearer TJ morphology relative to the DSS group, suggesting maintenance of their structural integrity. Compared with the control group, the DSS group showed a significant increase in FITC‐dextran 4000 levels, reflecting heightened colonic epithelial permeability and intestinal barrier damage. Leonurine treatment, however, ameliorated intestinal permeability (Figure 2E). The levels of TNF‐α and IL‐1β in leonurine group were significantly lower than in the DSS group (Figure 2F,G).

Figure 2Leonurine protects the intestinal barrier and decreases the levels of proinflammatory cytokines in DSS‐induced colitis models. (A) Immunohistochemical staining of claudin‐1 and occludin (scale bar: 100μm). (B) Electron micrographs images of colon tissues in each group. The red arrows indicate TJ (scale bar: 1μm). (C) Percentage of claudin‐1 positive cells in each group. (D) Percentage of occludin positive cells in each group. (E) Intestinal permeability assay. (F) TNF‐α in the serum of each group. (G) IL‐1β in the serum of each group. Values were plotted as mean ± SEM, *n* = 6.  ^∗^ *p* < 0.05,  ^∗∗^ *p* < 0.01,  ^∗∗∗^ *p* < 0.001.

**Figure:**
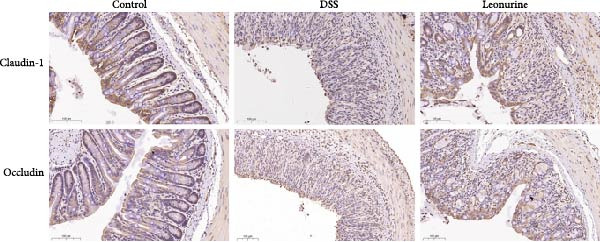
(A)

**Figure:**
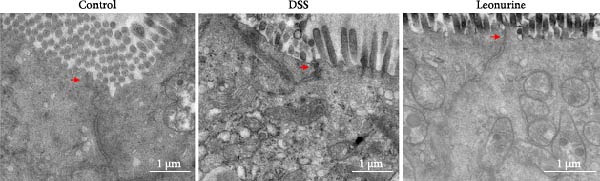
(B)

**Figure:**
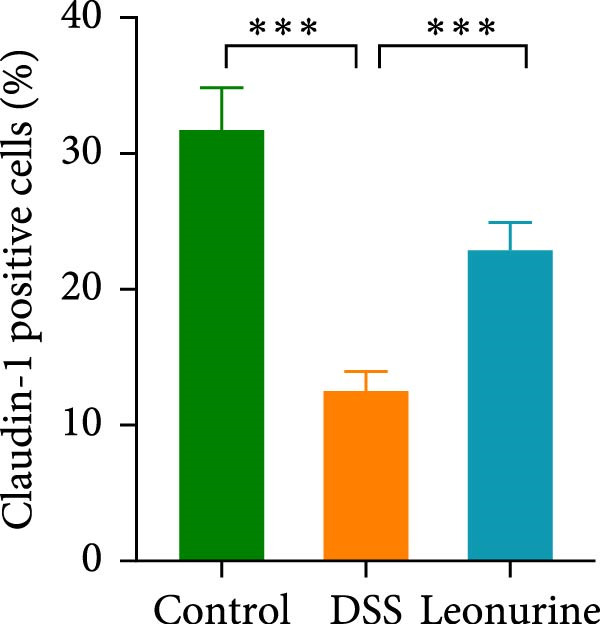
(C)

**Figure:**
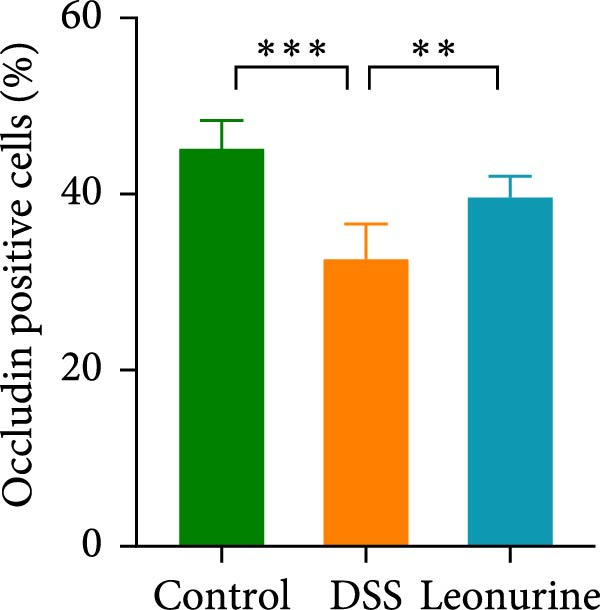
(D)

**Figure:**
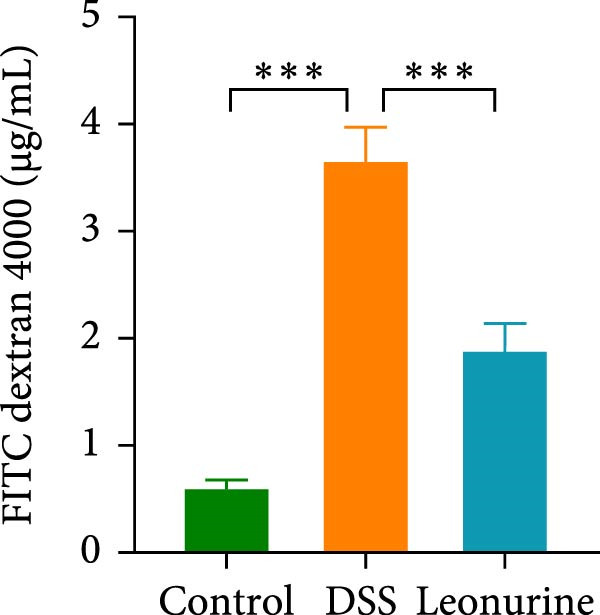
(E)

**Figure:**
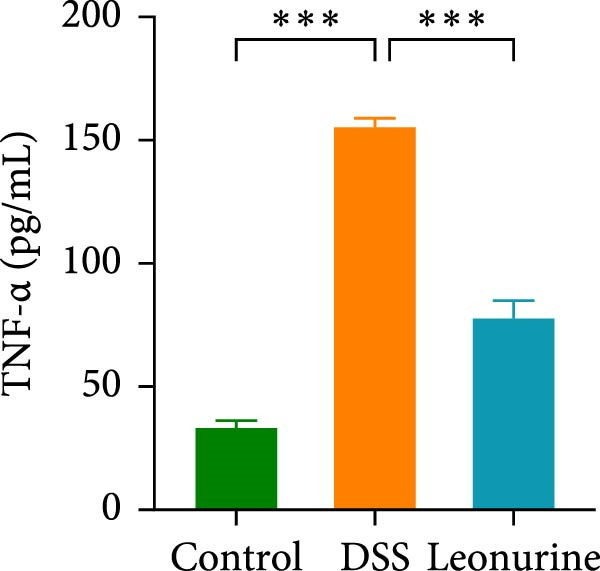
(F)

**Figure:**
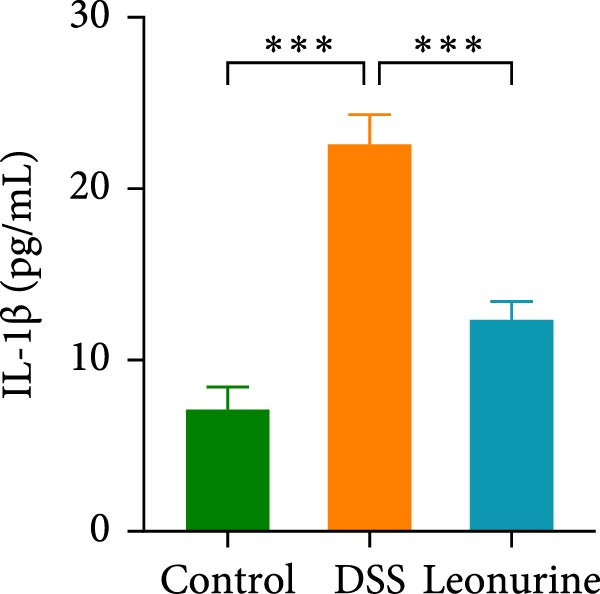
(G)

### 3.3. The Influence of Leonurine on the Transcriptome of the Colon

To further explore the potential mechanisms by which leonurine improved DSS‐induced colitis, we employed transcriptome sequencing of colon tissues from the control, DSS, and leonurine groups (*n* = 4 per group). A volcano map of the mice RNA‐sequencing results showed that the DSS group exhibited 4318 DEGs compared with the control group, with 2705 upregulated DEGs and 1613 downregulated DEGs (Figure 3A). A total of 861 DEGs were identified between the leonurine and DSS groups, of which 186 were upregulated and 675 were downregulated (Figure 3B). To screen the genes that met the treatment effect for further verification, we selected 452 DEGs that were upregulated in the DSS group and downregulated in the leonurine group and 70 DEGs that were downregulated in the DSS group and upregulated in the leonurine group (Figure 3C). Next, these DEGs were analyzed by GO and KEGG pathway enrichment (Figure 3D,E). We extracted the top 10 enriched GO terms including BP, cellular component (CC), and molecular function (MF). In GO BP terms, the DEGs were mostly enriched in “leukocyte chemotaxis” and “cell chemotaxis.” In GO CC terms, the DEGs were mostly enriched in “collagen‐containing extracellular matrix” and “secretory granule.” In GO MF terms, the DEGs were mostly enriched in “endopeptidase activity” and “peptidase inhibitor activity.” In total, two KEGG pathways were significantly enriched, including the protein digestion and absorption pathway and the pancreatic secretion pathway.

Figure 3The influence of leonurine on the transcriptome of the colon. (A) Volcano map of the differentially expressed genes between Control and DSS group. (B) Volcano map of the differentially expressed genes between Leonurine and DSS group. (C) Venn diagram of the differentially expressed genes among the three groups. (D) Dotplot of GO enrichment analysis. (E) Dotplot of KEGG pathway enrichment analysis.

**Figure:**
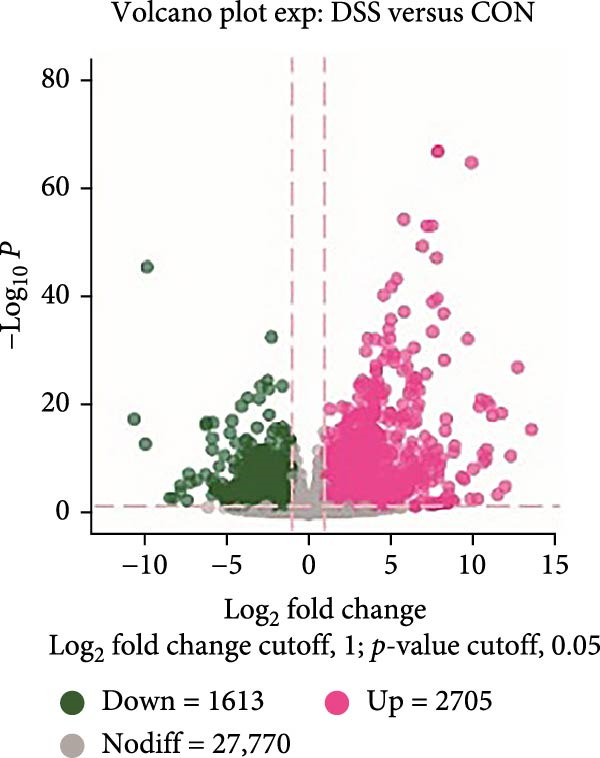
(A)

**Figure:**
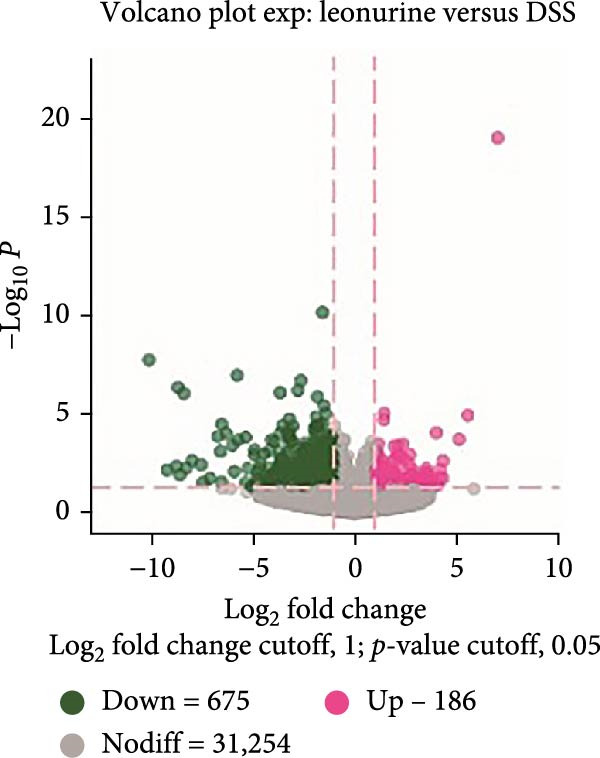
(B)

**Figure:**
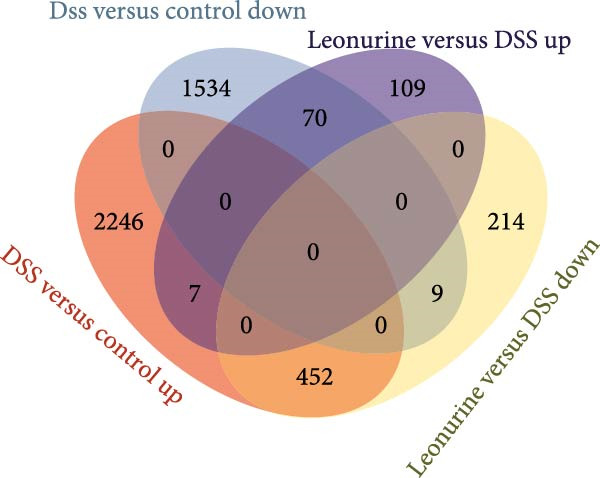
(C)

**Figure:**
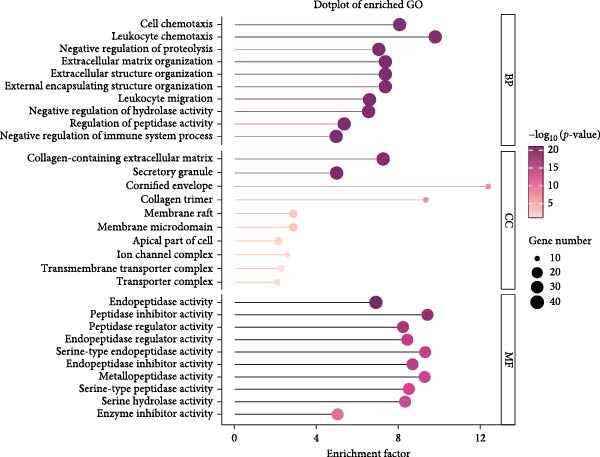
(D)

**Figure:**
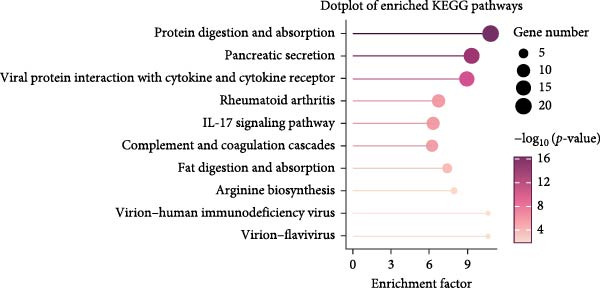
(E)

### 3.4. Leonurine Decreases Cela2a and Cela3b Expression in DSS‐Induced Colitis Models

In recent years, a growing body of evidence has shown that pancreatic exocrine function affects the gut immunity and the regulation of intestinal inflammation and homeostasis [16, 17]. Therefore, we listed 20 DEGs in the pancreatic secretion pathway, according to *p*‐values <0.05 (Figure 4A). Leonurine inhibited the expression of two chymotrypsin‐like elastase genes, *Cela2a* and *Cela3b*, in this pathway. This was reflected by significantly lower levels in the leonurine group compared with the DSS group. To verify the reliability of RNA sequencing, quantitative real‐time polymerase chain reaction (qRT‐PCR) was performed. The data indicated that the levels of *Cela2a* and *Cela3b* were increased by DSS compared with the control group, but lowered by leonurine compared with the DSS group (Figure 4B,C). The qRT‐PCR results regarding these two genes agreed with the RNA sequencing data.

Figure 4Leonurine decreases the expressions of *Cela2a* and *Cela3b* in DSS‐induced colitis models. (A) The heatmap shows the mean expression values of the top 20 DEGs associated with the pancreatic secretion pathway, derived from four samples per group (*p* < 0.05). (B) The expression of *Cela2a* mRNA in the colon tissue of each group was measured by qRT‐PCR. (C) The expression of *Cela3b* mRNA in the colon tissue of each group was measured by qRT‐PCR.  ^∗^ *p* < 0.05,  ^∗∗^ *p* < 0.01,  ^∗∗∗^ *p* < 0.001.

**Figure:**
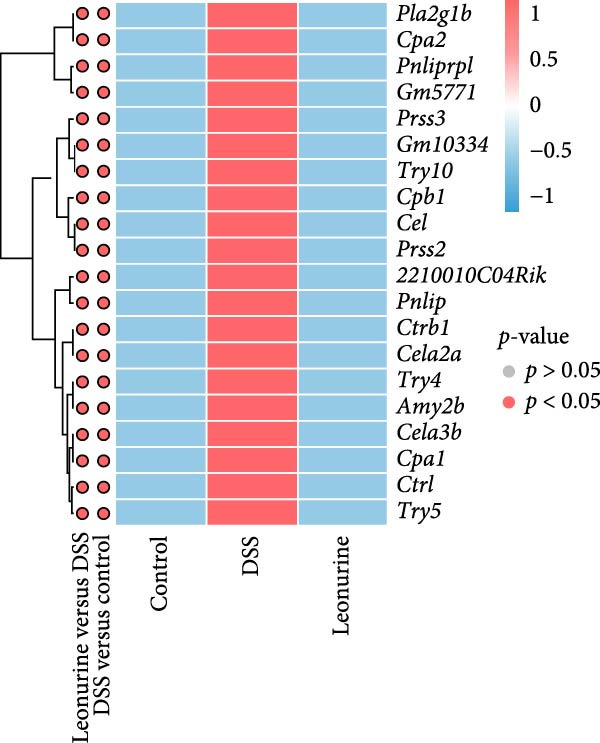
(A)

**Figure:**
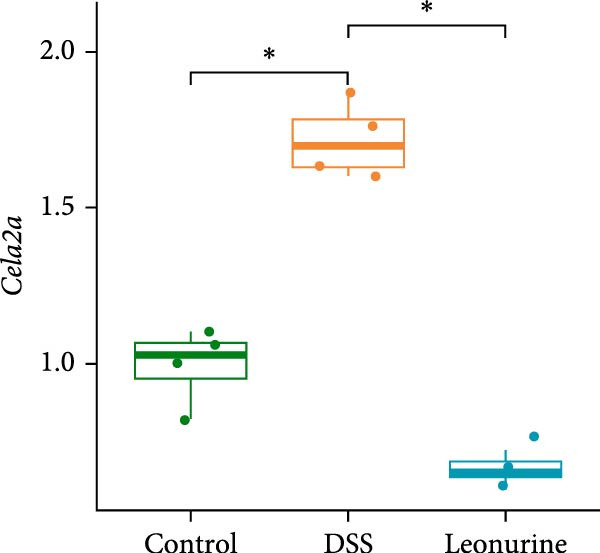
(B)

**Figure:**
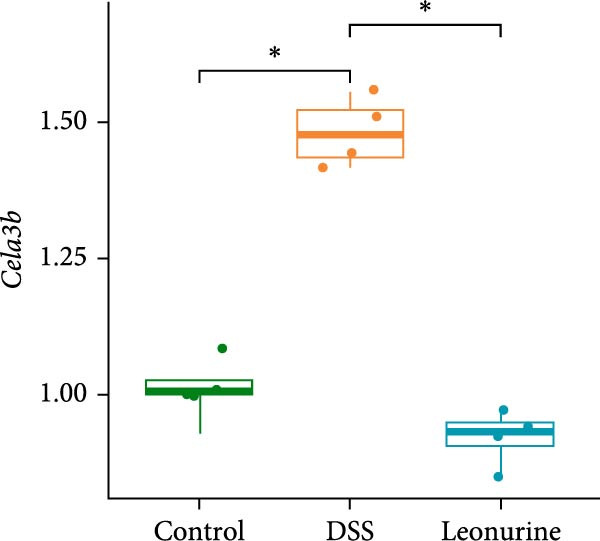
(C)

### 3.5. Leonurine Improves the Gut Microbiota of Mice

Fecal samples were analyzed to elucidate the influence of leonurine on the gut microbiota. A total of 733,973 raw sequences were generated through 16S rRNA gene sequencing of the three groups of mice. After splicing and filtering the raw data, 647,640 clean sequences were obtained. As shown in Figure 5A, the rarefaction curves tended to be flat, indicating that the depth and amount of the sequencing data in this study were reasonable. The length of good‐quality sequences was mainly distributed in the range of 400–440 bp, consistent with the length of the V3‐V4 region of the 16S rRNA gene sequences (Figure 5B). The sequencing results indicated that the data could be used for subsequent analysis. To evaluate the diversity differences between the three groups, the constrained principal coordinates analysis (PCoA) and PCoA method were performed to analyze the β‐diversity of the gut microbiota of mice from each group. There were significant differences in the gut microbiota between the three groups (Figure 5C,D). We used the Shannon index and Simpson index to assess the diversity of the microbial community (Figure 5E,F). We found that compared with the control group, the diversity of the microbiota of the DSS group decreased, with the decrease in the leonurine group being even more significant. At the phylum level, the colonic microbiota was dominated by *Bacteroidetes*, *Firmicutes*, and *Verrucomicrobia* (Figure 6A). At the genus level, Lachnospiraceae_NK4A136_group, *Muribaculaceae*, Clostridia_UCG‐014, Prevotellaceae_UCG‐001, and *Turicibacter* were the predominant genera (Figure 6B). In addition, Rikenellaceae_RC9_gut_group, UBA1819, *Enterococcus*, *Oscillibacter*, and *E*ubacterium_coprostanoligenes_group, were increased by DSS, but significantly reduced by leonurine treatment (Figure 6C).

Figure 5Between‐groups microbial diversity comparisons. (A) The rarefaction curves of all the samples. (B) Distribution of clean tags across samples. (C) Constrained PCoA analysis of the gut microbiota data of mice from each group. (D) PCoA analysis of the gut microbiota data of mice from each group. (E) Shannon index among the three groups. (F) Simpson index among the three groups.

**Figure:**
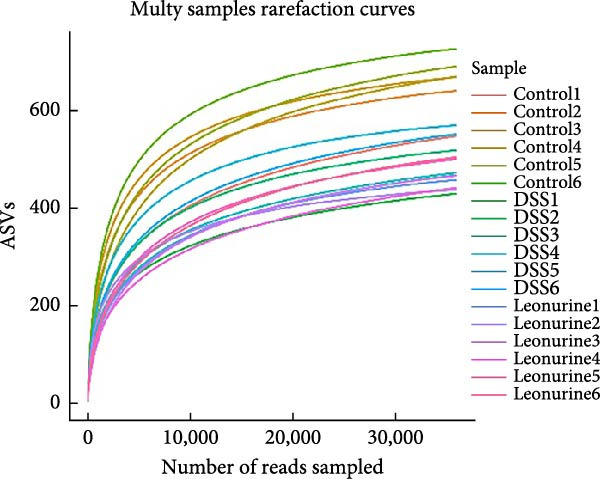
(A)

**Figure:**
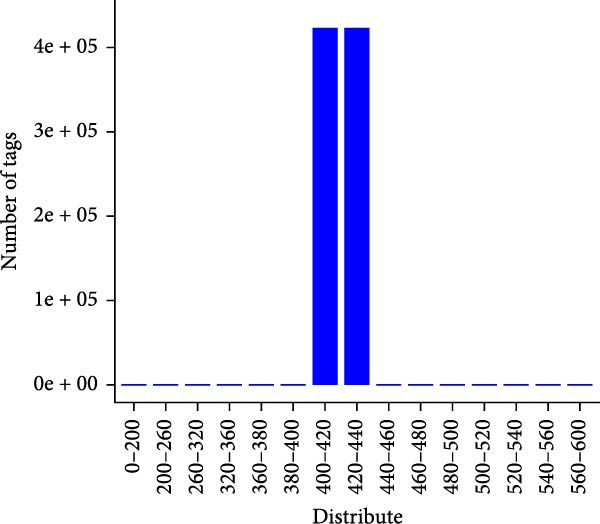
(B)

**Figure:**
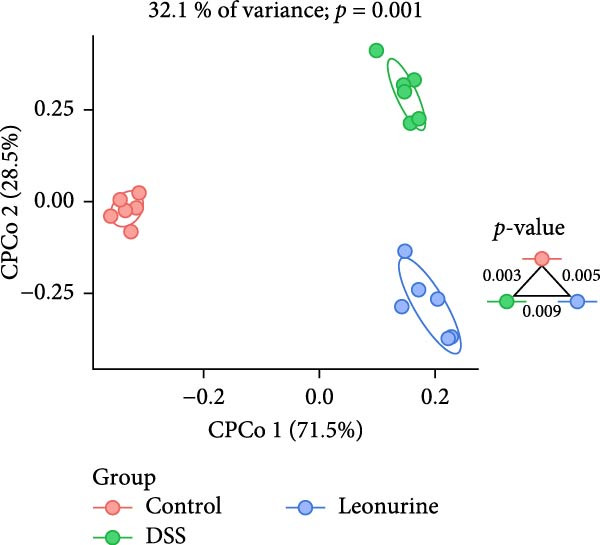
(C)

**Figure:**
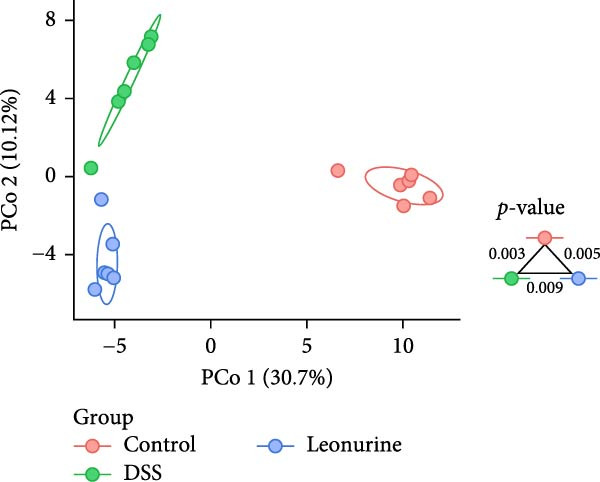
(D)

**Figure:**
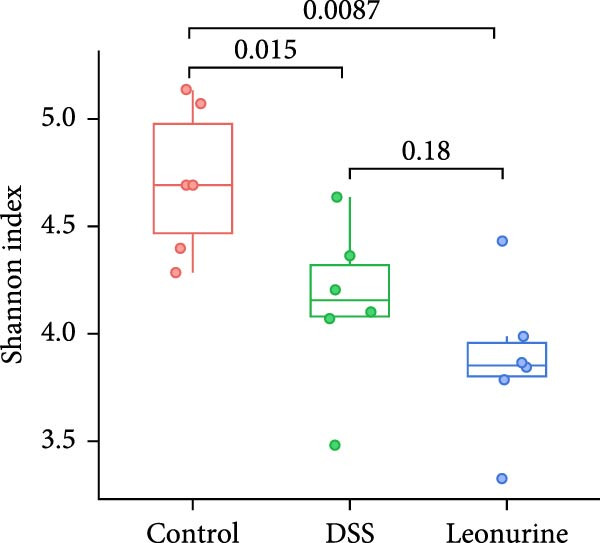
(E)

**Figure:**
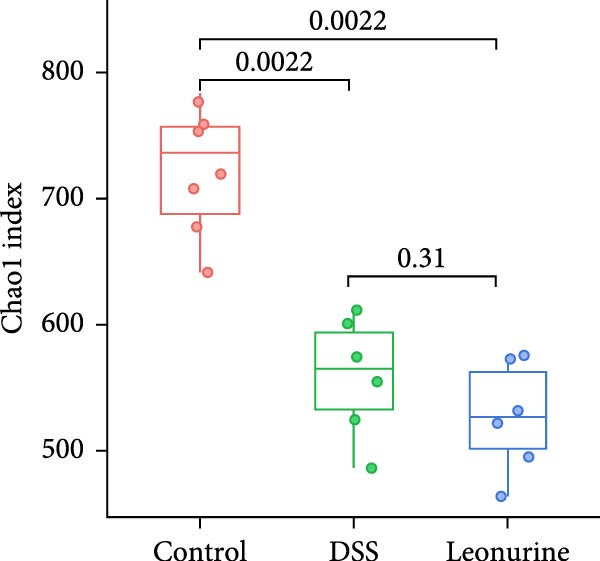
(F)

Figure 6Leonurine improves gut microbiota of mice. (A) Relative abundance in phylum level. (B) Relative abundance in genus level. (C) Differential test heatmap. (D) The relationship between the gut microbiota and the transcriptome data.  ^∗^ *p* < 0.05,  ^∗∗^ *p* < 0.01,  ^∗∗∗^ *p* < 0.001.

**Figure:**
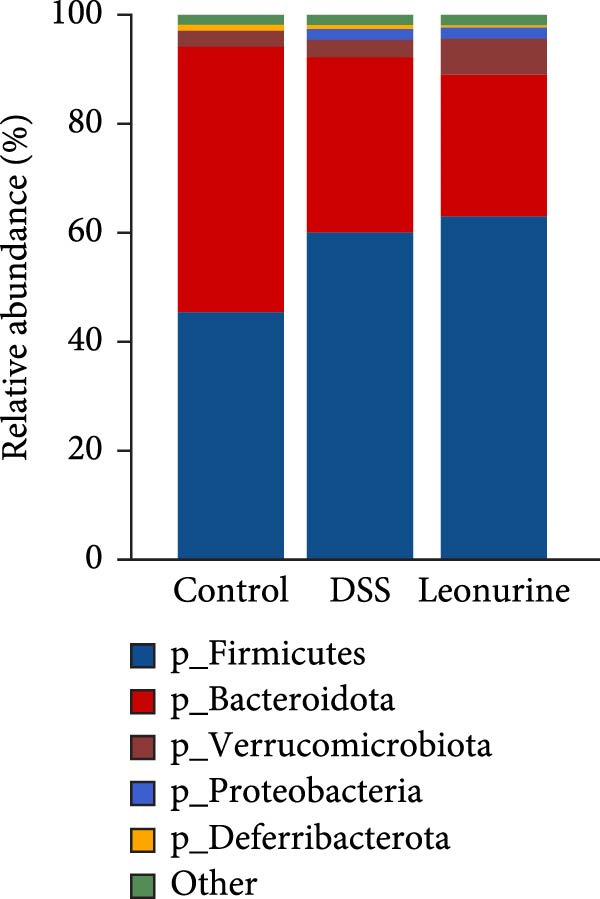
(A)

**Figure:**
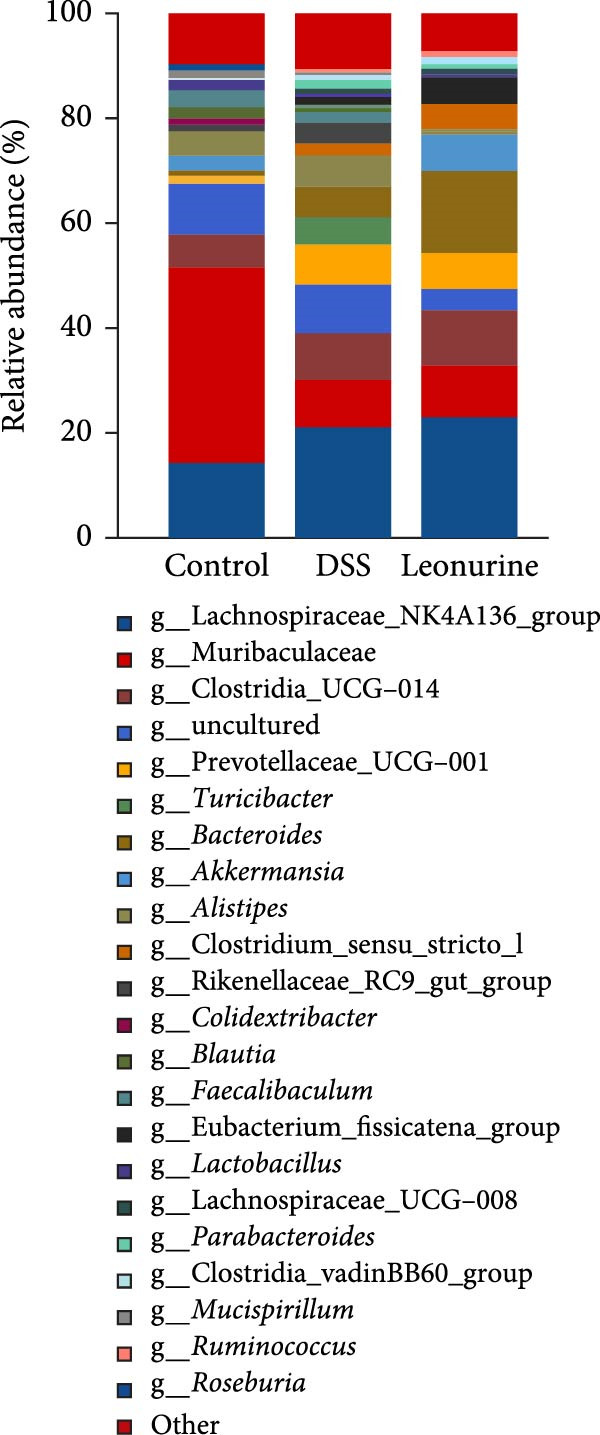
(B)

**Figure:**
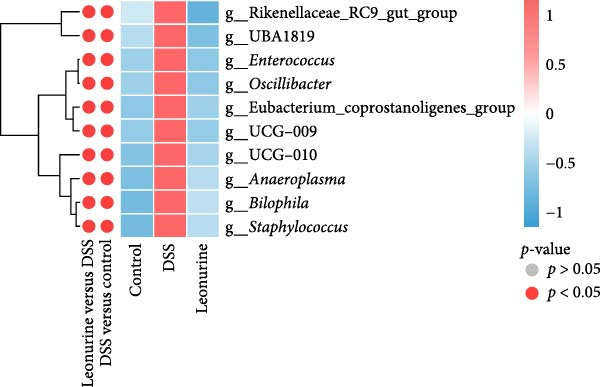
(C)

**Figure:**
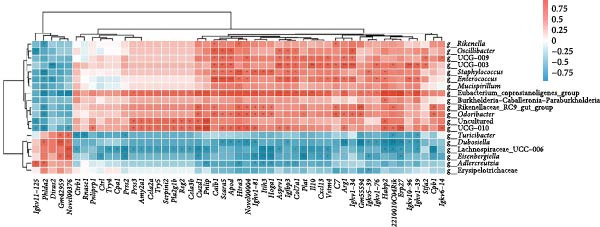
(D)

## 4. Disccusion

The pathogenesis of UC is multifactorial, involving epithelial barrier defects, genetic susceptibility, immune dysregulation, and other factors. Research into effective drugs for treating UC is increasingly garnering attention. Natural alkaloids have unique advantages in the treatment of UC due to their multitargeted effects and safety profile. Leonurine is a specific alkaloid found in the *Leonurus* genus. Increasing evidence indicates that leonurine has the effect of inhibiting oxidative stress and chronic inflammation. Leonurine regulates the Hippo pathway via miR‐21/YOD1/YAP, alleviating arthritis in mice by reducing joint inflammation and bone destruction [18]. It reverses acute liver injury by mitigating necrosis, inflammation, and oxidative stress [19]. It can also exert antiinflammatory and antioxidant effects by blocking the activation of the NF‐κB pathway, thereby providing effective cardiac protection against myocarditis [7]. The results in this study showed a protective effect of leonurine on the acute colitis in DSS‐induced UC mice model. Leonurine improved the inflammatory cells infiltrations, the body weight and colon length, and decreased the DAI score.

The two main proinflammatory cytokines associated with UC are TNF‐α and IL‐1β, which are markedly elevated in DSS group, and overexpression of TNF‐α and IL‐1β can lead to increased intercellular TJs permeability [20]. A defective TJ barrier allows intestinal bacteria and luminal antigens penetration, which accelerate the inflammation progression [21, 22]. In our study, we observed that leonurine treatment significantly decreased the levels of TNF‐α and IL‐1β and increased the expressions of claudin‐1 and occludin TJ proteins in DSS‐induced colitis. Findings from TEM and intestinal permeability assessment experiments indicated that leonurine ameliorates the intestinal barrier. The beneficial effect of leonurine on UC in mice may be attributed to its protective effect on the intestinal barrier by inhibiting the inflammatory response and enhancing the expression of TJ protein.

Transcriptome sequencing showed that the pancreatic secretion pathway was implicated in leonurine‐treated colitis as compared to the DSS group. The pancreatic secretion pathway is a vital signaling pathway that can induce pancreatic acinar cells to secrete digestive enzymes, and it’s important for the development of the body and digestive system [23]. Pancreatic duct ligation could against the gut barrier failure [24]. So proteases also play a pivotal role in maintaining gastrointestinal homeostasis and intestinal barrier [25, 26]. Many literature reports that a disbalance between proteases and protease inhibitors in the intestine contributes to gastrointestinal diseases such as irritable bowel syndrome, IBD, and celiac disease [27, 28]. The qRT‐PCR validated that leonurine downregulated the expression of *Cela2a* and *Cela3b* in DSS‐induced colitis. *Cela2a* is expressed in isolated human colon crypts, and compared to healthy controls, its expression is significantly increased in colonic UC tissues. This finding is in line with our results. *Ela2a*, the protein translated by *Cela2a*, was also overexpressed in UC tissues. Epithelial *Ela2a* hyperactivity could lead to intestinal barrier function loss, upregulation of proinflammatory mediators, and downregulation of antiinflammatory factors [29]. *Cela3b* is a pancreatic enzyme with digestive function in the intestine, expressed almost exclusively in the pancreas [30]. *Cela3b* has been related to pancreatitis and pancreatic adenocarcinoma [31]. Although the transcriptome sequencing suggested that *Cela2a* and *Cela3b* were involved in UC, the current research results mainly provide clues at the gene expression level. The functional studies have not kept pace. The specific mechanisms by which leonurine regulates the functions of *Cela2a* and *Cela3b*, how these functional changes further affect pancreatic secretion, and how these effects ultimately relate to the occurrence and development of colitis are yet to be thoroughly investigated. In the future, we need to further elucidate and refine the specific mechanisms by which leonurine influences colitis through the pancreatic secretion pathway by employing methods such as protein level detection, cell function experiments, and even pathway intervention experiments.

The gut microbiota is mainly composed of *Firmicutes, Bacteroidetes, Actinobacteria, Verrucomicrobia*, and *Proteobacteria* [32]. The microbiota provides benefits to the host in several ways, including host defense [33], nutrition, and immunity. However, an imbalance in the composition of the gut microbiota, known as dysbiosis, can disrupt these homeostatic functions. This dysbiosis is a typical feature of UC, and can cause damages to the intestinal microbial barrier [34]. Beyond the gut, such microbial alterations can also exert systemic effects; for instance, they have been linked to changes in brain function in patients with Parkinson’s disease via the gut‐brain axis [35]. In our study, compared with the control group, the relative abundance of *Firmicutes* in the DSS group increased, while that of *Bacteroidota* decreased. Although leonurine did not reverse this trend, it increased the relative abundance of *Verrucomicrobia* which has anti‐inflammatory and immunostimulant effects and can improve gut barrier function, endotoxinemia, and insulin sensitivity [36]. In this study, leonurine significantly reduced the abundance of *Oscillibacter*, Rikenellaceae_RC9_gut_group, UBA1819, and *Enterococcus*, exerting a protective effect on DSS‐induced colitis in mice. *Oscillibacter* is a key bacterial genus associated with IBD [37], contributing to increased intestinal permeability and negatively correlated with transepithelial resistance in the proximal colon [38]. A higher abundance of *Oscillibacter* in the gut microbiota may be associated with severe colitis induced by DSS [39]. The significant increase in the abundance of Rikenellaceae_RC9_gut_group has been shown to increase the sensitivity of the gut to inflammation [40]. The decrease in the abundance of UBA1819 helps maintain a healthy gut microbiota balance [41]. This may also have contributed to the reduced species diversity following leonurine treatment.

## 5. Conclusion

Leonurine ameliorates UC in mice by decreasing the levels of TNF‐α and IL‐1β, regulating the pancreatic secretion pathway, reducing the expressions of *Cela2a* and *Cela3b*, improving intestinal barrier integrity, and modulating the gut microbiota. However, the direct mechanism of action of leonurine on UC remains unclear and needs additional studies for clarification.

## Conflicts of Interest

The authors declare no conflicts of interest.

## Author Contributions

TingTing Cao and Ying Wang conceived, designed the experiments, and wrote the paper. TingTing Cao, Ying Wang and Juan Zhang performed the experiments. Wei Song and WeiJie Dai analyzed the data. GuoZhong Ji critically reviewed the manuscript. TingTing Cao and Ying Wang contributed equally to this work.

## Funding

No funding was received for this manuscript.

## Data Availability

The transcriptome data of this research were deposited into the NCBI Sequence Read Archive (SRA) database (Accession Number: PRJNA1124237).
